# Management Options for *Ixodes ricinus*-Associated Pathogens: A Review of Prevention Strategies

**DOI:** 10.3390/ijerph17061830

**Published:** 2020-03-12

**Authors:** Jiří Černý, Geoffrey Lynn, Johana Hrnková, Maryna Golovchenko, Natalia Rudenko, Libor Grubhoffer

**Affiliations:** 1 Faculty of Tropical AgriSciences, Czech University of Life Sciences in Prague, Prague 165 00, Czech Republic; hrnkova@ftz.czu.cz; 2 Section of Infectious Diseases, Department of Internal Medicine, Yale University School of Medicine, New Haven, CT 06420, USA; geoffrey.lynn@yale.edu; 3 Institute of Parasitology, Biology Centre, Czech Academy of Sciences, České Budějovice 370 05, Czech Republic; marina@paru.cas.cz (M.G.); natasha@paru.cas.cz (N.R.); liborex@paru.cas.cz (L.G.)

**Keywords:** tick management, tick, *Ixodes ricinus*, tick-borne diseases

## Abstract

Ticks are important human and animal parasites and vectors of many infectious disease agents. Control of tick activity is an effective tool to reduce the risk of contracting tick-transmitted diseases. The castor bean tick (*Ixodes ricinus*) is the most common tick species in Europe. It is also a vector of the causative agents of Lyme borreliosis and tick-borne encephalitis, which are two of the most important arthropod-borne diseases in Europe. In recent years, increases in tick activity and incidence of tick-borne diseases have been observed in many European countries. These increases are linked to many ecological and anthropogenic factors such as landscape management, climate change, animal migration, and increased popularity of outdoor activities or changes in land usage. Tick activity is driven by many biotic and abiotic factors, some of which can be effectively managed to decrease risk of tick bites. In the USA, recommendations for landscape management, tick host control, and tick chemical control are well-defined for the applied purpose of reducing tick presence on private property. In Europe, where fewer studies have assessed tick management strategies, the similarity in ecological factors influencing vector presence suggests that approaches that work in USA may also be applicable. In this article we review key factors driving the tick exposure risk in Europe to select those most conducive to management for decreased tick-associated risk.

## 1. Ecological Drivers Affecting Tick Activity

**Figure 1:**
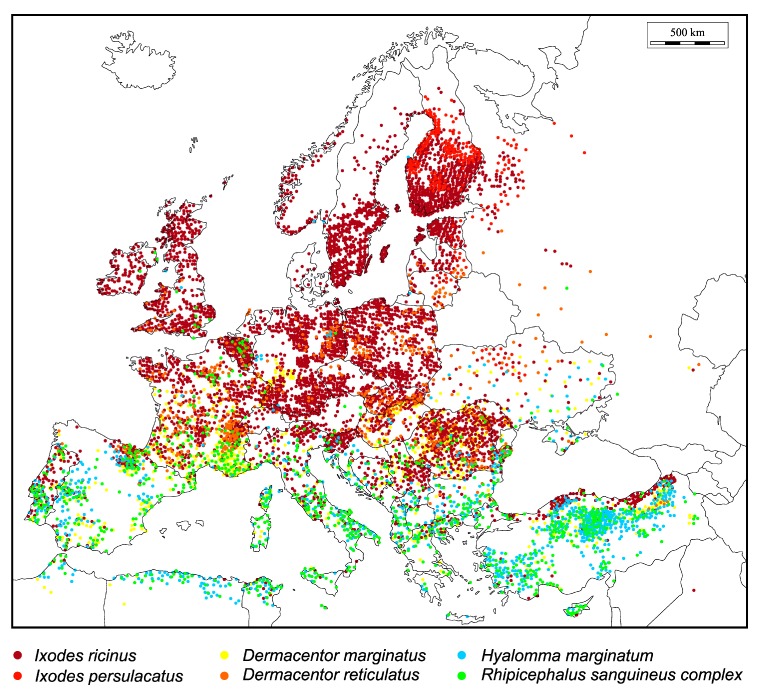
Ticks in Europe: Distribution of six medically and veterinary important tick species in Europe is depicted. The figure shows geographic locations where important species have been reported (as indicated by colored dots) and was prepared according to data from Estrada-Peña et al. [1].

Ticks (Ixodida) are the most important vectors of arthropod-borne pathogens in Europe. Out of more than 900 currently described known tick species, approximately 10% are of medical importance to humans [1,2]. In Europe, approximately 70 tick species are established [1]. European tick species are classified within two families and seven genera, namely: *Dermacentor*, *Haemaphysalis*, *Hyalomma*, *Ixodes*, and *Rhipicephalus* (all Ixodidae), and *Argas* and *Onithodoros* (both Argasidae). As a part of the VectoNet project, the European Centre of Disease Prevention and Control (eCDC) monitors seven tick species (*Ixodes ricinus*, *Ixodes persulcatus*, *Dermacentor reticulatus*, *Hyalomma marginatum*, *Rhipicephalus bursa*, *Rhipicephalus sanguineus*, *Ornithodoros* spp.) due to their potential to transmit important human pathogens [3]. Some of tick species, such as *Dermacentor marginatus*, are vectors of important animal diseases [4]. The most important tick vectors in Europe are widely distributed throughout the continent (Figure 1). *Ixodes ricinus* is the most abundant European tick species [1] and therefore, this review will focus on this tick. 

*Ixodes ricinus* is a vector for multiple tick-borne diseases common in Europe and as a result has a significant impact on public health. [5]. It is the primary vector for *Borrelia burgdorferi* sensu lato spirochetes, causing Lyme borreliosis, and tick-borne encephalitis virus. Further, many other important tick-borne pathogens (TBPs) such as *Borrelia miyamotoi*, *Rickettsia slovaca*, *Rickettsia helvetica*, *Rickettsia monacensis*, *Anaplasma phagocytophilum*, *Babesia divergens*, *Babesia venatorum*, *Babesia microti*, *Bartonella henselae*, *Coxiella burnetii* and *Francisella tularensis* have been detected in *I. ricinus* and it is either confirmed or likely that this tick species is an important vector for many of these TBPs [6,7]. The active lifecycle of *I. ricinus* includes three developmental stages: larva, nymph, and adult. As a three-host tick, during each of these stages the tick must obtain a single blood meal which allows it to molt and advance to the subsequent stage, or for adult females, to successfully oviposit. Larval and nymphal feeding events offer opportunities for ticks to acquire TBPs from potentially-infected hosts. As most TBPs are either not transmitted transovarially, or have low transovarial transmission efficiency, later developmental stages are generally more likely to carry TBPs [8]. Although *Ixodes ricinus* males may feed on vertebrates to some extent, their role in TBPs transmission is considered minimal. It is however, possible they may influence TBPs ecology via sexual transmission of some tick-borne viruses from infected males to naïve females, as was previously reported in other tick species [9,10]. 

*Ixodes ricinus* is present over a broad geographic range, from the Mediterranean coast of north Africa on its southern edge, to the Arctic circle at the northern limit, and from Portugal in the west to Karelia, the Baltic states, and Ukraine in the east, where it shares its biotopes with a closely related tick *I. persulcatus* [1,3]. *Ixodes ricinus* is most frequently observed in lowland habitats, though it has been found on animals at much greater elevations up to 2000 meters above sea level (a.s.l.) in Switzerland [11] (these animals were most likely infested at lower altitudes). Field collection of ticks in Czechia and Austria demonstrated that questing *I. ricinus* of all developmental stages could be found at localities about 1300m a.s.l., though their prevalence rapidly decreased according to increase in altitude [12,13,14,15]. In accordance with climate change trends, *I. ricinus* ticks are reported more frequently at higher elevations and new foci are emerging north beyond the previous extent of their range [14,16,17], resulting in an expanded region where people are at risk for tick-borne diseases (TBDs). Taken together, tick activity and prevalence of TBPs are steadily growing in Europe in recent years due to increased activity within long-standing, well established foci, as well as expansion into newly emerging zones [18,19,20,21,22].

*Ixodes ricinus* is an exophilic tick that actively quests for hosts and consequently, its presence is strongly associated with specific biotope and climatic conditions. Typical habitats where *I. ricinus* are present tend to be lowland, relatively humid biotopes such as unmanaged grasslands, heaths, forest edges, woodlands, and broad-leaf forests with sufficiently dense undergrowth [23]. Key climatic factors influencing tick activity include air and soil temperature, air and soil humidity, and solar radiation [21,24,25,26]. Additionally, climatic cycles associated with seasonality have a major impact on host-seeking period and diapause. In central Europe, ticks are typically active within the period from March/April to October/November [27], but as they can be active in temperatures close to freezing point, adult ticks can be occasionally found questing during warmer winter days [26]. The lowest relative air humidity limit in which *I. ricinus* has been recorded active is 24% [28]. At these lower limits of tolerable humidity, following brief periods of active questing, relocation to the base of vegetation/soil interface to rehydrate is required for ticks to avoid desiccation [28]. For rehydration, *I. ricinus* requires relative humidity in the microhabitats close to soil to be approximately 85% [29]. In drier conditions *I. ricinus* is not able to survive and this intolerance for low humidity is an important limitation preventing *I. ricinus* from establishing populations in arid regions near the Mediterranean [24]. For this same reason, *I. ricinus* activity in central Europe normally peaks in May/June and in September, which are relatively warm and humid months [21,30,31]. Given that climate, and precipitation in particular are predicted to become more irregular in Europe, it is likely that tick activity will change correspondingly in the future [18]. 

Another very important ecological determinant for *I. ricinus* is the presence of suitable hosts. *Ixodes ricinus* does not typically aggregate on hosts in large quantities and therefore it does not harm them by blood loss connected with feeding itself like other tick species (e.g., *Amblyomma americanum* or *Rhipicephalus microplus*) can [32]. *Ixodes ricinus* has a very wide range of potential hosts including mammals, birds, and reptiles. Larvae and nymphs feed preferentially on small mammals (rodents, insectivores), birds, and reptiles, while adult females prefer medium size or large mammals (lagomorphs, carnivores, and ungulates). However, the latter set of hosts can also host immature stages [33,34]. Questing ticks were shown to be more frequently present in localities with conspicuous signs of host presence such as wildlife trails and bedding sites, relative to surrounding terrain [35]. Moreover, because *I. ricinus* ticks are ambush predators and consequently, do not actively move long distances, dispersal by animal hosts, especially migratory birds is an important vehicle for establishing new populations of *I. ricinus* [36,37].

The *I. ricinus* life strategy as a host generalist is very important with respect to transmission of TBPs. On one hand, this offers the opportunity to infect many host species. However, not all of these species may be efficient reservoir hosts for pathogens for one of several reasons [38] including transient presence of viremia/bacteremia/parasitemia in the hosts [39,40], low pathogen concentration in host tissues and/or blood, or active host immune response against pathogen [41,42]. Host immunity can even lead to clearance of pathogen not only from the host, but also from the tick itself as in the case of borreliacidal activity of lizard serum [43,44]. 

## 2. Anthropogenic Factors Affecting Tick Activity and Abundance of Tick-borne Diseases

While important, environmental factors are not the only factors leading to increased activity of ticks and elevated prevalence of tick-borne diseases. In addition, human alterations to landscapes and changes in human activities that increase exposure to tick inhabited biotopes can amplify TAR.

Several studies have shown that human induced changes in local biotopes that lead to greater abundance of tick hosts also result in increased tick activity and prevalence of TBPs [12,34,45]. Conversely, activities such as forest clearance can lead to decreased tick activity [46]. Effect of grazing on TAR is ambiguous. Grazing can lead to decrease of tick activity as it leads to change in vegetation cover which become more hostile to ticks, but it also provides more potential hosts for tick feeding which could lead to increased TAR [47,48]. The use of insecticides in agriculture can have a significant effect on tick densities. Randolph et al. hypothesized that decrease in agricultural intensity following collapse of Soviet empire in eastern Europe at the beginning of 1990s, was one of the factors leading to increase of tick-borne disease prevalence in these countries [49]. However, this theory could be challenged on the basis that agriculture in eastern European countries has recovered during past 20 years with regard to intensity as well as return to insecticide usage [50,51], yet the incidence of TBDs continues to increase [52].

Epidemiological data also show that incidence of tick-borne diseases increases during periods of economic instability and that low-income economic classes are more vulnerable to this increase [49]. Examples include the prohibitive cost of vaccination against TBEV as well as an increased reliance on activities based in areas where risk of tick exposure is elevated [49]. Furthermore, participation in outdoor leisure activities in high risk areas for tick exposure is one of the key variables determining number of reported tick bites and prevalence of TBPs. It was shown that number of tick bites reports increased during sunny and warm conditions following rainy periods, when more people travel to forested areas for leisure activities, such as hiking, and picking forest berries or mushrooms [53]. Similarly, calendar peaks in nymphal activity and TBEV incidence frequently coincide with peak of mushroom picking period in countries where this activity is a popular leisure activity or a source of occasional income [52,54].

## 3. Tick Management Actions

**Figure 2:**
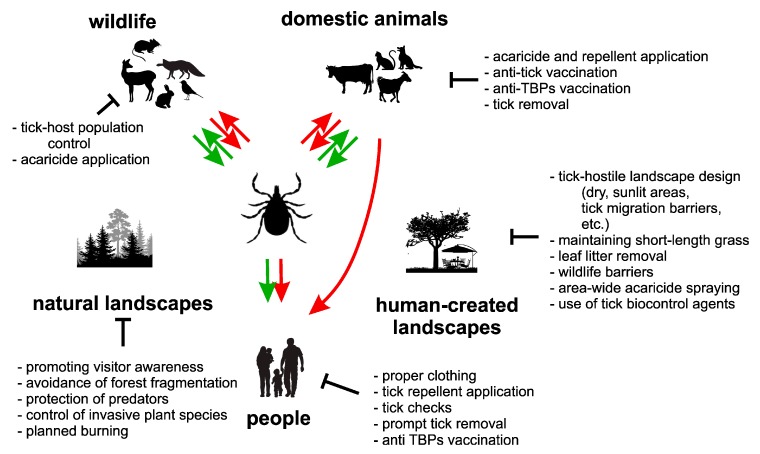
Tick management strategy: Tick activity and risk associated with TBPs can be managed using a broad variety of approaches. These include direct reduction of tick populations using chemical or natural-origin acaricides, tick pathogens (e.g., fungi), or encouraging tick predators. Further, tick activity can be decreased by limiting availability of potential animal hosts (e.g., rodents or ungulates) and by creating tick-hostile environments (dry, sunlit areas lacking leaf litter and other vegetal debris) which limit tick survival and reproduction. Finally, TAR can be decreased by adhering to proper practices (wearing long clothes, using tick repellents, checking body after visiting tick-infested areas and prompt removal of all attached ticks) as well as basic public health (vaccination against TBEV, medical consultation immediately after appearing of any possible marks of infection by a TBP). Green arrows show pathways important in tick life cycle; red arrows show possible avenues of TBP transmission.

Tick-associated risk (TAR), defined here as the potential for exposure to pathogens via tick bite, is heavily influenced by both tick and human activity. Therefore, evidence-based management of these activities can lead to decrease of TAR (see Figure 2 for graphical description of TAR management actions). 

To date, the subject of tick management has been studied more extensively in North America than it has been in Europe. Therefore, for certain applications and methods where data from Europe is lacking, the authors of this review are limited to projecting the effect of these measures based on observations in North America. The observed outcomes in North America may not be mirrored in Europe precisely as many conditions (e.g., tick species, host species) are different. In Europe (north of the Alps), *I. ricinus* is the most medically-important tick species as it accounts for vast majority of TBP infections [1]. In North America the *Ixodes* ticks (*I. scapularis* and *I. pacificus*) are also major source of TBP infections, but they are more often accompanied by other ticks from different genera (e.g., *Amblyomma*, *Dermacentor*, *Rhipicephalus*, etc.) which can expand the variety of TBPs present in a given location [55]. Further, Europe and North America belong to different biogeographic zones (Palearctic and Nearctic, respectively). It brings important differences in many aspects such composition of potential host species (e.g., small mammals) as well in vegetation cover. But the minor differences in fauna and flora mentioned earlier, would only create minor differences in TAR.

### 3.1. Personal Protective Measures

Personal protection is a specifically targeted, and often, the most practical approach for tick prevention. It includes reducing time spent in tick infested habitats, using the adequate tick protective clothing and repellents, and physically checking oneself for ticks after leaving the tick infested habitats [56]. 

Ticks are typically not distributed evenly across the landscape and are more often concentrated in habitats with suitable microclimate conditions and higher concentrations of their hosts [57,58]. Therefore, when hiking in natural habitats, it is advisable to avoid shaded places with dense undergrowth and attempt to use areas with open canopies and minimal grass coverage [57,58]. Human use of wildlife trails also increases the likelihood of tick encounter [35]. When hiking through tick-infested localities, one should walk down the middle of the pathway and minimize contact with surrounding vegetation [56]. 

Wearing light-colored clothes is beneficial as it makes ticks easier to detect and remove before they have the opportunity to attach. Long pants tucked into socks and wearing non-open shoes can reduce contact with ticks [56,59]. 

Repellents can also dramatically decrease the risk of tick attachment [60,61] and for optimal benefit, proper application of repellents should include consideration for the concentration of active compound and the duration of activity. Modern synthetic anti-tick repellents which can be applied directly on the human skin frequently contain N,N-diethyl-*m*-toluamide (DEET), ethyl butylacetyl-aminopropionate (IR3535) or 1-piperidinecarboxylic acid (picardin), as primary active ingredients. Permethrin-based repellents (3-(2,2-dichloroethenyl)-2,2-dimethylcyclopropanecarboxylic acid, (3-phenoxyphenyl) methyl ester) should be limited to treatment of clothing as the chemical is a skin irritant. The potential for carcinogenic activity has been demonstrated for rats [60,62], but has not confirmed for humans for permethrin [63]. Naturally occurring compounds offer another option and have been used as repellents for many years [64]. Currently, numerous compounds isolated from plant essential oils are being tested and used for their tick repellent activity and they show promising results [65,66,67,68,69]. Moreover, these compounds are considered not to be harmful for humans or ecosystems. Various products are already sold commercially as potential tick repellents, though effectiveness of some of them has yet to be scientifically proven.

Careful inspection of one’s entire body after returning from tick infested environment, followed by proper and prompt removal of all found ticks is another important behavior that can help prevent of TBD [56]. Though, some pathogens (e.g., TBEV) are transmitted shortly after tick attachment, others (e.g., *Borrelia burgdorferi* sensu stricto or babesia apicomplexans) need up to several days of feeding before being transmitted. Attached ticks should be removed using tweezers or a tool similarly capable of grasping the tick as closely as possible to its mouthparts [70]. 

### 3.2. Vaccination, Prophylaxis and Treatment

The only vaccine(s) for TBD currently available for human usage are the vaccine against tick-borne encephalitis virus [71]. A vaccine against Lyme borreliosis approved for human usage was available in USA between 1998 and 2002 but was later withdrawn from the market [72,73]. However, vaccines against Lyme borreliosis are still available for veterinary usage [74,75]. Several research teams are currently working on development of other Lyme borreliosis vaccines, and more broadly, anti-tick vaccines, which could impede tick feeding and interfere with transmission of multiple tick-borne diseases. This approach has previously been applied successfully with BM86 derived vaccine against the cattle tick *Rhipicephalus microplus* [76,77].

For *B. burgdorferi*, it has been demonstrated that a single 200 mg dose of doxycycline within 72 hours after a bite by infected tick can prevent onset of Lyme disease [78,79]. Doxycycline treatment can be in this case offered as an evidence-based treatment option as ticks can be commercially tested for presence of Borrelia and results may be available within 24 hours from submission. Among the antibiotics effective against tick-transmitted bacterial infections, doxycycline is the primary option for treatment of Lyme borreliosis and anaplasmosis and is most effective if used during the early stages of infection) [80,81]. Tick-borne piroplasmida are sensitive to quinine derivates [80,82]. Currently there is no anti-viral drug to cure the infections caused by tick-borne viruses, and therefore treatment in these cases is limited to supportive care. However, several compounds have been proven to inhibit TBEV replication in mice in laboratory experiments [83] and may progress to viable treatment options in the future.

### 3.3. Tick Host Management

As obligate parasites, ticks are dependent on the presence of appropriate hosts. The same is true for TBPs, which in general, are enzoonotically maintained. Therefore, suitable management of tick hosts can lead to decrease of both ticks and TBPs.

Mathematical modeling and in field experiments done in the USA have shown that abundance of the North American tick *Ixodes scapularis*, a close relative of *I. ricinus*, is strongly associated with density of deer [84]. A decrease in numbers of deer was consistently associated with a decrease in tick population in following years [85,86]. Field studies in Europe yielded similar results for *I. ricinus* [87,88]. Nevertheless, these studies still need further confirmation [89]. Because population control of large ungulates by hunting is not feasible, it would be interesting to test whether the presence of ungulate predators (e.g., wolves or lynxes) might have an impact on tick activity [90]. 

Small vertebrates, especially rodents, are important reservoirs of most TBPs, which makes them potential targets for prophylactic approaches. In one such application of this strategy, placing of acaricide containing rodent bait boxes led to decrease in Lyme borreliosis prevalence USA [86]. Similarly, providing the rodents with acaricide-treated cotton for nest lining could lead to decrease in TBDs in treated areas [91]. *Ixodes ricinus* abundance also depends on rodent abundance. Increased activity of rodent predators led to a decrease in tick abundance and a decrease in prevalence of TBP-infected ticks. [92]. Nevertheless, as *I. ricinus* immature stages are not exclusively dependent on small mammals, the potential effect of bait boxes on their populations has yet to be determined at broad scale. Further, deer can be protected from tick-infestation by acaricide application using special “4-Poster” feeding racks that apply acaricide to the head and neck regions as the deer access the feed. One study reported that these devices were associated with a rapid decrease in tick activity in areas studied. [93]. Nevertheless, widespread use of “4-Posters” is complicated because of many factors which include the high price for installation and maintenance, unknown effects on deer and other animals, and potentially, the human health impacts of long term exposure to acaricides contained in the “4-Posters” [94].

In regions with low numbers of wild ungulates but high numbers of livestock, livestock can play a role of the host on which adult tick females feed and it can also serve as reservoir of TBPs [95]. Contrary to livestock, companion animals such as cats and dogs do not play a significant role in ecology of *I. ricinus* or *I. ricinus*-borne diseases. These are usually just occasional hosts for *I. ricinus* and dead-end hosts for *I. ricinus*-borne pathogens. Nevertheless, companion animals also suffer from TBDs. For example, infection of dogs by *Borrelia* spirochetes can lead to arthritis [96]. 

Apart from those measures which are used also for humans, several additional approaches can be used to decrease TAR for non-human animals. This includes both the anti-tick BM-86-based vaccine working against *Rhipicephalus* spp. but not *Ixodes ricinus* [97] and a vaccine to prevent Lyme disease in canines [98]. Additionally, various acaricides can be applied directly on animal skin, though most require multiple applications over the course of a tick season [99,100].

Finally, it is important to mention possible direct transmission of some TBPs from infected animals to humans by contaminated animal product. The best-known example in Europe is transmission of TBEV from small ruminants to humans by unpasteurized milk, leading to a massive outbreak in Rožňava (Slovakia) in 1951 that resulted in the infection of an estimated 660 persons [101]. Direct transmission between infected animals and humans has also been proven for other TBPs such as Omsk hemorrhagic fever virus [102]. Infection with TBPs can also occur through blood transfusions [103,104].

### 3.4. Landscape Management

Because *I. ricinus* presence is limited by highly specific habitat requirements, carefully planned landscape management can easily lead to significant decrease of TAR. Such landscaping actions should be preferentially targeted to localities with high frequency of human outdoor activities during tick activity season such as city parks, recreational venues, sporting areas, campsites, and other outdoor areas. 

Tick management can be challenging in protected environments such as nature reserves, where efforts are made to minimize impact of human activities in these areas. In these instances, the focus should be on informing and promoting visitor awareness of TARs in order to successfully influence behavioral patterns (see previous sections) [56]. Additionally, minimally-invasive actions such as cutting grass short, or leaf litter removal can be performed at minimum, around the visitor centers and the most frequent hiking paths [56]. Planned burning of some localities as a part of fire management has also proven to be effective for management of ticks on other continents [105,106] though data from Europe are absent and planned burns are not currently allowed in most European countries. Furthermore, legislation intended to decrease TAR can be implemented. For example, this might include restrictions designed to prevent forest fragmentation, which can lead create favorable habitat for preferred hosts of certain tick species, including *I. ricinus* [107,108,109]. Additionally, for certain areas, livestock grazing might offer a cost-effective compromise between productive land use and limiting TAR. Intensive grazing by domestic animals has been shown to decrease TAR in multiple studies [47,48].

In human-altered landscapes such as public parks, stronger measures can be employed relative to natural landscapes. Residential lawns were shown to be poor environments for *I. scapularis* [110], and the reduction of tree canopy cover, thereby increasing sunlight penetration and decreasing humidity, can lead to further reduction in tick activity and survival. Migration of ticks to the maintained areas can be decreased by installing > 1 m wide wood chip, mulch, sand or gravel border between lawn and tick infested areas [111]. Using spaces of this kind that are unwelcoming to ticks for leisure activities such as barbecues etc. could decrease TAR. Likewise, whenever possible, children’s playgrounds should be placed in these tick-hostile zones. 

Tick management can provide benefits not only in natural and suburban settings, but also in urban areas [112]. For example, city parks have been shown to support high tick densities as well as high prevalence of tick-borne pathogens [113]. Additionally, synanthropic animals have been shown to be in frequent contact with TBPs and could thereby potentially serve as reservoirs of these diseases in urban settings where ticks are also present [114]. Nothing is known about tick activity on brownfields and other abandoned postindustrial localities, which are also part of urban landscape. 

As most of tick bites in Europe are reported to occur in publicly accessible areas, which typically account for larger areas than privately owned lands, the primary responsibility to employ adequate anti-tick landscape management lies with local municipalities or districts. Therefore, it is paramount that informed citizens take an active role lobbying their local government agencies and national public health authorities to invest resources toward decreasing TAR. Sufficient financial support is often necessary as these efforts are typically costly and require long-term sustained action to produce measurable results [115]. Fortunately, some of these actions minimizing TAR are being successfully employed in Europe, such as increased percentage of TBEV vaccinated citizens [116], while other solutions, such as increase in the percentage of grazed land are achieved as a part of other landscape management goals [117].

### 3.5. Acaricides

Acaricides (pesticides that kill ticks and mites), are widely used for tick management and are a particularly effective tool for livestock producers to limit *Rhipicephalus microplus* infestation of cattle [118,119]. Acaricides are also an effective option for managing *I. scapularis* ticks [120]. Nevertheless, several tick species from genus *Rhipicephalus* have already developed resistance against many acaricides [121,122]. 

The most commonly used acaricides currently are chlorinated hydrocarbons (e.g., lindane), organophosphorus compounds (e.g., coumaphos), carbamates (e.g., carbaryl), formamidines (e.g., amitraz), pyrethroids (e.g., permethrin, flumethrin), formamidines (e.g., amitraz), macrocyclic lactones (e.g., ivermectin), phenylpyrazoles (e.g., fipronil), insect growth regulators (e.g., fluazuron), and isoxazolines (e.g., afoxalaner, fluralaner, sarolaner). These compounds differ with regard to route of application, length of protection, efficiency, specificity, ability of ticks to reach resistance and many other parameters [119,123]. There are yet additional acaricidal options beyond those listed here, which we will not include given both the broad scope of this review, and existence of reviews that cover the subject in greater depth [62,124].

Theoretically, acaricides can be used in two different ways. They can be applied either topically on potential tick hosts, an application that is typical for protection of livestock and companion animals [125]. Alternatively, acaricidal formulations can be sprayed broadly in a liquid formulation, where an area, rather than a host is being treated. This type of application has been demonstrated to be very effective for controlling tick populations on private properties in the USA [120]. Nevertheless, effective acaricidal broad-spraying requires that specific conditions are met including appropriate temporal period of application, favorable weather conditions during application, and typically, use of several rounds of re-application. Therefore, it is often prohibitively expensive in many instances and may only feasible for smaller areas. Moreover, many acaricides are toxic for insects, beneficial mites, and/or aquatic organisms. In Europe, reports of decreased insect abundance and diversity at both the individual, and the species levels [126]. Therefore, the authors of this review feel that proper acaricide broad-spraying should be recommended only on very limited scale and in absence of other effective options. Currently, novel eco-friendly compounds isolated from natural plant sources with acaricidal activity are being tested with promising results [119,127,128]. Nevertheless, these substances may also impact non-target species, such as ecologically-beneficial arachnids and should evaluated for such outcomes prior to large-scale application.

### 3.6. Biological Agents

Several biological agents have been described that could potentially be used to manage tick populations. A minute parasitic wasp, *Ixodiphagus hookeri*, is a geographically widespread tick parasite. The adult females of this wasp lay their eggs into the ticks of many genera (*Ornithodoros, Amblyomma, Dermacentor, Haemaphysalis, Hyalomma, Ixodes and Rhipicephalus*). Unfortunately, field studies done in New England, where *I. hookeri* was artificially introduced, showed that its presence does not have a significant impact on local tick populations [129]. 

Ticks are also known to be parasitized by several nematode species [130]. In nature, nematodes from family Mermethidae were found parasitizing ticks [131]. Under laboratory conditions nematodes from families Steinernematidae and Heterorhabditidae showed robust ability to kill *Rhipicephalus* ticks [130].

Perhaps the most promising pathogens of ticks are two fungal species, *Metarhizium anisopliae* and *Beauveria bassiana* [132]. They showed not only a strong capability to kill ticks in the laboratory and field studies, but also to negatively impact tick populations by reducing the reproductive efficacy of survivors [133]. Several strains of them are currently approved for area-wide use in management of ticks [134].

Ticks are also preyed upon by many soil-dwelling arthropods such as predatory mites, spiders, and ants [135]. Engorged ticks of all stages are especially prone to predation [135]. According to anecdotal observations, fowls such as chickens, guinea-fowls, and turkeys are able to reduce local tick populations. The best described tick predators are African oxpeckers, which specialize on various exoparasites of large mammals [136]. One study showed that oxpecker presence was negatively correlated with tick abundance [137]. Additionally, some species of rodents and insectivores are reported to actively feed on ticks [138]. Paradoxically, these animals also serve as very important tick hosts, so their presence could both limit and increase tick density. 

Biological control is used for management of mosquitoes and mosquito-borne pathogens and may provide guidance for tick-related applications. This includes two strategies which are very promising in management of mosquito associated risks, yet understudied in TAR management research. First, the impact of midgut microbiome on mosquito vector competence has been studied extensively [139,140,141]. Several laboratory and field experiments showed that release of *Wolbachia*-infected mosquitoes can reduce prevalence of mosquito-borne diseases such as Zika, dengue, and chikungunya [142]. *Wolbachia* have also been identified in *A. americanum*, yet their effect on vector capacity of this tick is not known [143]. Likewise, other symbionts have been described for a range of tick species and several have been shown to affect pathogen transmission, including a *Rickettsia* endosymbiont of *Dermacentor andersoni* which can facilitate competitive exclusion of a pathogenic species (*R. rickettsia*), and a Coxiella endosymbiont of *A. americanum* that may impair transmission of a pathogen that causes ehrlichiosis (*Ehrlichia chaffeensis*) [144,145], Yet with a few exceptions, the interactions between most microbes and ticks are poorly understood and in general, our understanding of the relationship between tick microbiome and vector capacity is in its early stages [146].

Secondly, genetically modified mosquitoes, especially those bearing mutations causing male sterility or female lethality are widely and successfully utilized in field trials to manage mosquito populations [147]. While this strategy is also a priority for tick-control, ongoing research is still in developmental stages and practical application is therefore unlikely to be an option available for tick management in the short-term. 

## 4. Tick Management in the USA in Comparison to Europe

As in Europe, TAR is a significant public health concern in the USA. However, there are several important differences potentially influencing TAR which influence how it should be managed. In Europe, most exposures to TBPs occur during outdoor activities in public areas. Whereas in high incidence parts of the northeastern USA where suburban residential properties are frequently located in wooded, tick-infested areas, most infections are acquired peridomestically, on private properties [148,149]. In the latter instance, it can be assumed that individuals have greater opportunity to actively manage the environments where they are most likely to be exposed, whereas persons in Europe may have less influence over conditions in settings where they may be exposed, and thereby, greater reliance is placed on the role of local government. 

Secondly, in the USA, it is relatively uncommon for livestock to graze in forested environments where *I. scapularis* ticks are present. However, in Europe, recent changes such as more intensive livestock production, and related land usage practices such as grazing in outfield (minimally managed) pastures can lead to increased interaction between *I. ricinus*, livestock and humans [150,151]. Balancing economic interests with public health creates an added degree of complexity these locations.

On the other hand, many ecological drivers affecting tick activity and associated prevalence of TBPs are very similar in Europe and the USA. Therefore, it can be expected that Europe could benefit from adopting approaches that are successfully employed in the USA to improve on current management of TAR. Perhaps the principle lesson to be learned from practices in the USA may be the opportunity to improve deficits in the overall population’s awareness about TAR, which would lead to better individual prevention as well as to emergence of new markets focused on management of TAR. In the USA, evidence-based, state-issued advisories for the public and healthcare professionals, related to decreasing TAR are available and typically easily accessible [111,152]. These can be useful tools for land owners to plan and manage their properties according to prescribed recommendations, often with the effect of creating markets for landscape architects, tick testing biotechnological companies, facility management companies and others. Currently, these types of recommendations are not available in most European countries and it is probable that an increase in awareness of strategies to limit TAR could lead to improved practices and a reduction in TBD. 

## 5. Conclusions

Ticks and TBPs pose a significant threat to humans and domestic animals in Europe. Current climatic and social changes have been linked to increases in TAR in areas where ticks have traditionally present, as well in new areas where novel foci are emerging. 

The most abundant tick in Europe is *I. ricinus*, and its presence is closely tied to biotope suitability, which includes sufficient humidity, vegetation cover, and the availability of animal hosts. TAR is connected not only with tick abundance but also with human activities in tick infested habitats, many of which can be managed to decrease tick-associated risk. 

Proper personal protective practices, beginning with appropriate clothing and use of tick repellents when entering tick infested habitats, as well as personal checks after leaving these areas are basic fundamental behaviors for avoiding tick bites. These measures can be applied not only for humans, but also for domestic animals. Management of wildlife hosts for ticks is also a potentially effective approach, which can include encouraging the presence natural predators of these tick hosts, therein limiting availability of hosts, and ultimately, reducing tick populations and prevalence of TBPs. Further, outdoor public spaces with high human activity during tick season (city parks, recreational spaces, nature preserves, etc.) should be properly managed to decrease TAR. This includes regular grass cutting or grazing, and removal of residual organic debris (cut and flattened grass, fallen leaves, etc.) Because most tick bites in Europe are associated with activities in public outdoor localities, management for TAR should be organized by local municipalities or districts. In cases when simple mechanical measures are not sufficient to decrease TAR, chemical treatment by acaricides can be employed, while carefully accounting for the potential toxicity of these chemicals and adhering to environmental regulations that exist to minimize potential risk for local ecosystems. Use of biological agents, which are much less harmful to other parts of local biotopes, is an alternative to chemical acaricides for controlling tick populations, but even these approaches can have a negative effect on beneficial, non-target arthropod species. Information about the impacts of predators on tick abundance in total, is very sparse and more research is needed [153]. Therefore, further research is needed to develop, test, and release new natural acaricides and improve their specificity. Manipulation of the tick microbiome and genetic modification to reduce vectorial capacity are two additional promising, yet currently under-developed approaches that may eventually be realized.

While there is currently no perfect single solution, TAR can be reduced using an integrated approach to tick management This type of approach has been shown to be very useful at reducing mosquito-borne infections, with the caveat that such programs require close management and long-term maintenance [87,154,155].
